# A New Equation to Estimate Muscle Mass from Creatinine and Cystatin C

**DOI:** 10.1371/journal.pone.0148495

**Published:** 2016-02-05

**Authors:** Sun-wook Kim, Hee-Won Jung, Cheol-Ho Kim, Kwang-il Kim, Ho Jun Chin, Hajeong Lee

**Affiliations:** 1 Department of Internal Medicine, Seoul National University Bundang Hospital, Seongnam, Republic of Korea; 2 Seoul National University College of Medicine, Seoul, Republic of Korea; 3 Graduate School of Medical Science and Engineering, Korea Advanced Institute of Science and Technology, Daejeon, Republic of Korea; 4 Department of Internal Medicine, Seoul National University Hospital, Seoul, Republic of Korea; University of Leicester, UNITED KINGDOM

## Abstract

**Background:**

With evaluation for physical performance, measuring muscle mass is an important step in detecting sarcopenia. However, there are no methods to estimate muscle mass from blood sampling.

**Methods:**

To develop a new equation to estimate total-body muscle mass with serum creatinine and cystatin C level, we designed a cross-sectional study with separate derivation and validation cohorts. Total body muscle mass and fat mass were measured using dual-energy x-ray absorptiometry (DXA) in 214 adults aged 25 to 84 years who underwent physical checkups from 2010 to 2013 in a single tertiary hospital. Serum creatinine and cystatin C levels were also examined.

**Results:**

Serum creatinine was correlated with muscle mass (*P* < .001), and serum cystatin C was correlated with body fat mass (*P <* .001) after adjusting glomerular filtration rate (GFR). After eliminating GFR, an equation to estimate total-body muscle mass was generated and coefficients were calculated in the derivation cohort. There was an agreement between muscle mass calculated by the novel equation and measured by DXA in both the derivation and validation cohort (*P* < .001, adjusted R^2^ = 0.829, β = 0.95, *P* < .001, adjusted R^2^ = 0.856, β = 1.03, respectively).

**Conclusion:**

The new equation based on serum creatinine and cystatin C levels can be used to estimate total-body muscle mass.

## Introduction

Aging leads to biological and physical changes in the structure and function of skeletal muscle. Sarcopenia has been defined as a phenomenon of age-related progressive decline in skeletal muscle mass and function that may result in decreased strength and low physical performance. It has been known that sarcopenia is associated with functional impairment, increased risk of fall down,[1] and consequently with decreased quality of life. Therefore, sarcopenia is assumed to be a major factor of geriatric syndromes and cycle of frailty. Moreover, sarcopenia is related to metabolic diseases (e.g. diabetes mellitus, dyslipidemia), major adverse cardiovascular events, and mortality.[2–4]

In detecting sarcopenia, algorithms required measuring physical performance or muscle strength and muscle mass.[5, 6] To date, many methods have been developed to measure muscle mass and diagnose sarcopenia. One of classical methods for estimating muscle mass is calculating 24-hour urinary creatinine excretion.[7] However, the reliability of this test is largely dependent on the subject’s compliance.[8] With technical improvement, methods for estimating muscle mass with computed tomography (CT) or magnetic resonance imaging (MRI) have been established, and currently they are considered the gold standards in research.[9, 10] Recently, dual-energy x-ray absorptiometry (DXA) and bioimpedance analysis (BIA) have been often used to estimate muscle mass in routine practice.[11, 12] Although imaging modalities like MRI, CT, and DXA are considered to produce precise results, these methods have caveats in terms of cost, possible radiation exposure, and limited accessibility for primary care and field studies. Furthermore, these tests cannot be performed using archived samples of serum in large scale cohorts.[13] On the other hand, BIA has its weakness in low precision and reproducibility especially in patients who have chronic illness or extreme body height or weight.[14, 15]

Assessment of glomerular filtration rate (GFR) is essential for clinical practice, research, and serum creatinine has been widely used for estimating GFR.[16, 17] However, creatinine-based GFR estimation is largely influenced by physiological and clinical conditions that affect muscle mass.[18] The serum creatinine levels of sarcopenic elderly are usually low or below normal ranges; therefore, estimated GFR (eGFR) calculated with serum creatinine level, usually overestimates their real kidney function.[19] Recently, cystatin C has been the focus of a new marker for GFR which is a low molecular weight protein produced with a stable production rate and filtered by the glomerulus freely.[20] Because it is not influenced by dietary factor or muscle mass, cystatin C-based eGFR is more appropriate for the elderly who are susceptible to sarcopenia.[21, 22]

We focused on the fact that cystatin C is independent of muscle mass, and hypothesized that discrepancy between creatinine and cystatin C-based GFR can be explained by the muscle mass. Therefore, we aimed to develop a novel equation to estimate total-body muscle mass (TBMM) with serum creatinine and cystatin C levels.

## Methods

### Participants

The current study is a retrospective cross-sectional study. We studied community-dwelling participants aged ≥25 years who visited the health promotion center of a single tertiary hospital for health screening from January 2010 to June 2013. Data were collected through an electronic medical record system. A total of 303 people who had done DXA as well as those who had undergone measurement of serum creatinine and serum cystatin C were screened. We excluded 85 people who underwent these tests over a month of period and 4 people who had metal prosthesis which interfere with measuring muscle mass with DXA. Finally, records of 214 people were included for the analysis, and none of included people had been diagnosed with chronic kidney disease. Each person was assigned 3-digit random number electronically, and categorized into two groups with 1:1 distribution by the random number. The first group served as an equation-derivation cohort and the second group as an equation-validation cohort. The study protocol was reviewed and approved by the institutional review board of the Seoul National University Hospital, which waived the requirement for informed consent.

### Measurement

Previous medical history was acquired, and clinical variables including height, weight were measured. All people included in the analysis underwent DXA, measurement of serum creatinine level, and serum cystatin C level within 30 days. TBMM and total-body fat mass were estimated using DXA (Lunar prodigy advance, GE healthcare, Fairfield, CT) with standardized protocols. TBMM was considered equivalent to the value of total-body lean mass minus total-body bone mass, assuming that nonfat and nonbone tissue was muscle.[23] Assays for serum creatinine and cystatin C were performed at the Seoul National University Hospital immediately after sampling. Serum cystatin C concentration was determined by using a particle-enhanced immunoturbidimetric assay (MODULAR P analyzer, Roche/Hitachi, Indianapolis, IN).

### Construction and Validation of the New Equation

**Fig 1:**
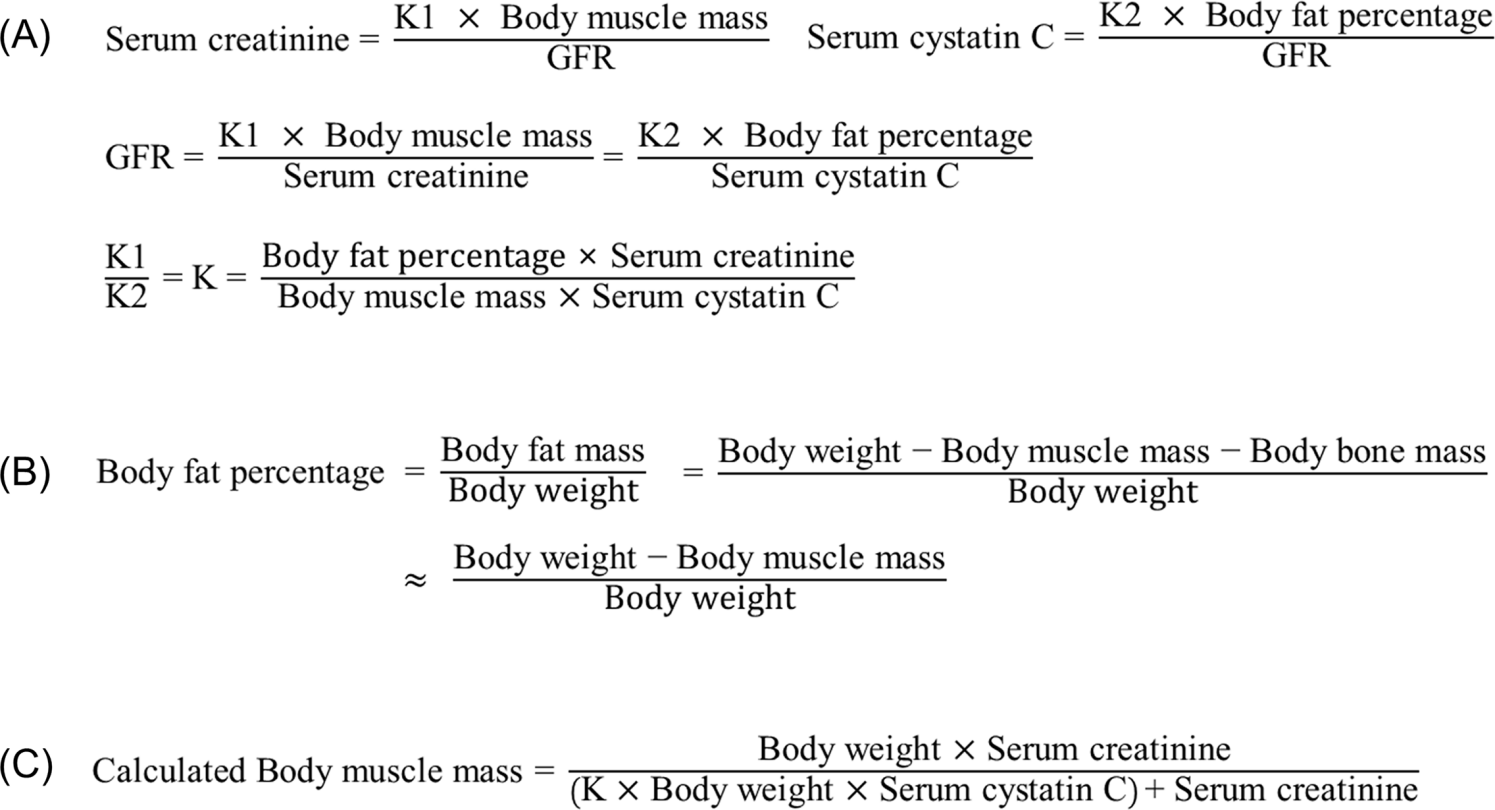
Development of the novel equation to estimate muscle mass.

We assumed that the serum creatinine level is proportional to the TBMM and inversely correlated to the eGFR. We also presumed, based on a previous study, that the serum cystatin C level is proportional to the total-body fat percent and inversely correlated to the eGFR.[24] We made a linear equation based on those suppositions then determined the coefficient K in the derivation cohort (Fig 1A). Total-body bone mass accounts for less than 5% of total-body muscle mass; therefore, we eliminated bone mass from the equation (Fig 1B).[25] After elimination of the eGFR and total-body fat percent, a final equation to estimate TBMM was developed (Fig 1C). Confirmation of the final equation was done in the validation cohort and performance was reported.

### Statistical Analysis

Continuous variables were expressed as mean (SD) and discrete variables were expressed as counts (percentages). Linear regression analysis was used to evaluate the association between cystatin C and total-body fat percent. The association between creatinine and TBMM was assessed. Performance of the novel equation was described by the R^2^ value and plotted in a scatter plot. We compared calculated TBMM and TBMM measured with DXA minus calculated TBMM with a Bland-Altman plot graphically.[26] To assess the relationships between TBMM by DXA and TBMM by the novel equation, we calculated intraclass correlation coefficients (ICC). We logged and analyzed data using PASW Statistics 18.0 (SPSS Inc., Chicago, IL) and MedCalc (Medcalc Software, Acacialaan 22, Ostend, Belgium). All records from participants were de-identified and analyzed anonymously.

## Result

One hundred and seven people were assigned to the derivation cohort and 107 were assigned to the validation cohort using a random number table. The baseline characteristics of the derivation cohort (37 men and 70 women) and validation cohort (34 men and 73 women) are shown in Table 1. There was no significant difference between two groups.

**Table 1:** Baseline characteristics of study participants.*

	Derivation cohort		Validation cohort	
	Men (n = 37)	Women (n = 70)	Men (n = 34)	Women (n = 73)
**Demographic parameters**				
**Age, y**	62.8 (9.3)	62.0 (9.9)	61.7 (9.6)	62.3 (10.5)
**Height, cm**	165.6 (6.2)	154.7 (6.3)	168.3 (6.5)	153.8 (5.7)
**Weight, kg**	69.7 (9.5)	59.4 (10.7)	74.0 (17.1)	58.2 (8.5)
**BMI, kg/m**^**2**^	25.4 (3.2)	24.8 (3.9)	26.0 (4.7)	24.6 (3.4)
**Laboratory parameters**				
**Creatinine, mg/dL**	1.00 (0.15)	0.74 (0.20)	0.98 (0.14)	0.72 (0.13)
**Cystatin C, mg/L**	0.83 (0.17)	0.80 (0.17)	0.81 (0.12)	0.78 (0.15)
**eGFR, mL/min/1.73m**^**2**^^†^	81.9 (12.9)	87.0 (15.7)	82.8 (11.9)	88.2 (14.7)
**Body composition with DXA**				
**Muscle mass, kg**	46.3 (4.5)	33.4 (4.1)	49.6 (8.3)	32.7 (3.8)
**Fat mass, kg**	18.3 (5.8)	21.6 (7.5)	18.9 (9.7)	21.3 (6.0)
**Bone mass, kg**	2.8 (0.4)	2.1 (0.4)	2.9 (0.5)	2.0 (0.3)

*Mean ± SD (all such values).

^†^Estimated by the CKD-EPI creatinine equation

Abbreviation: BMI-Body mass index, DXA-Dual-energy x-ray absorptiometry, eGFR-Estimated glomerular filtration rate

To verify hypotheses, we evaluated the relationship between the creatinine level and TBMM in the derivation cohort. There was a linear correlation between serum creatinine level and TBMM after adjusting for CKD-EPI creatinine equation eGFR (*P* < .001, R^2^ = 0.804). We also examined the relationship between the cystatin C level and total-body fat percent. After correction of CKD-EPI creatinine equation eGFR, there was a linear correlation between the serum cystatin C level and total-body fat percent (*P* < .001, R^2^ = 0.475).

**Fig 2:**
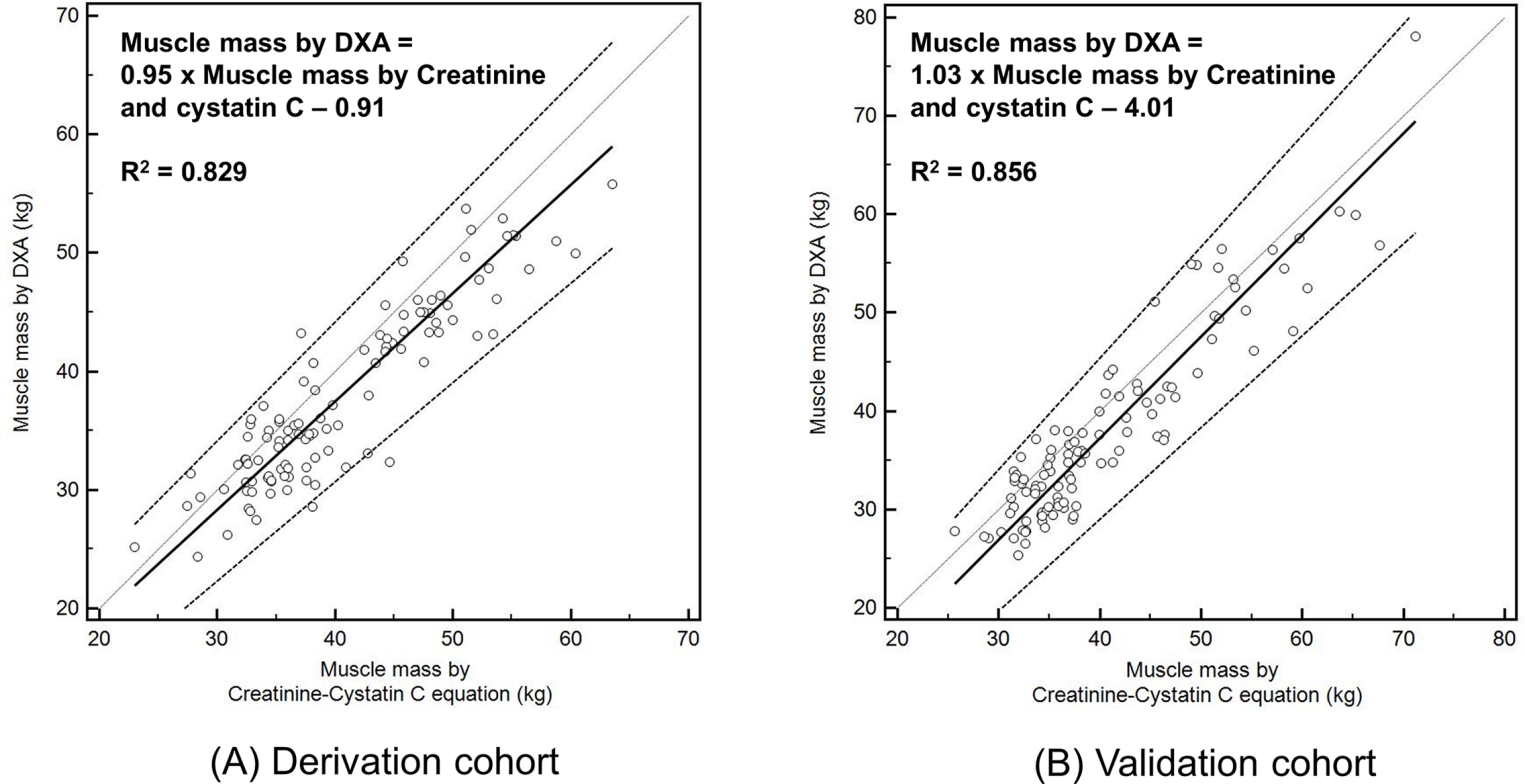
Scatter plots of muscle mass measured with DXA and the novel equation. Thick line indicates mean trend line and dotted line indicates 95% confidence interval. Legend: DXA- Dual-energy x-ray absorptiometry.

**Fig 3:**
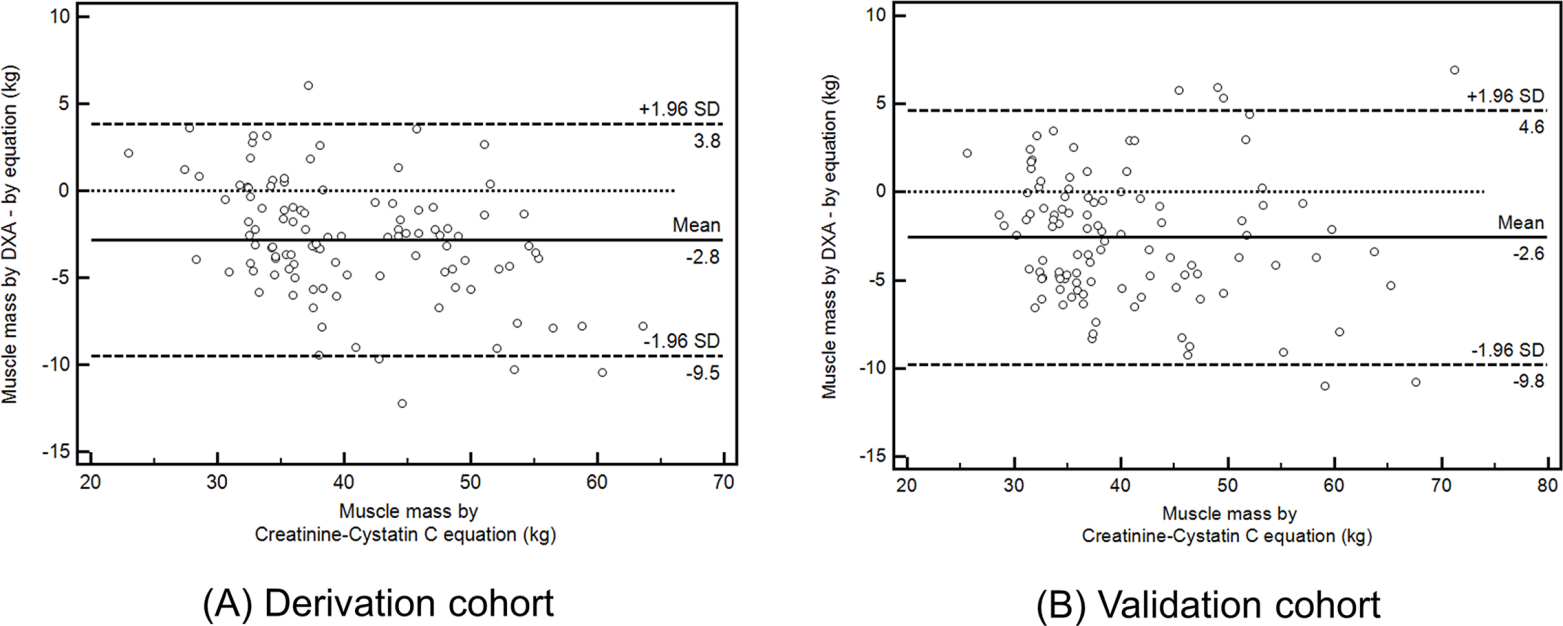
Bland-Altman plots of muscle mass measured with DXA and the novel equation. Legend: DXA- Dual-energy x-ray absorptiometry, SD-Standard deviation.

With serum creatinine level, serum cystatin C level, DXA measured total-body fat percent, and TBMM, we determined the coefficient K value in the derivation cohort (Fig 1A). We choose the mean value of the coefficient K for men and women separately, considering possible influence of sex on metabolisms of creatinine and cystatin C.[27] The coefficient values were 0.00675 for men and 0.01006 for women. We calculated individual muscle mass with the novel equation and determined coefficient values (Fig 1B), and the calculated TBMM explained 82.9% of the between-subject variance in DXA measured TBMM (*P* < .001, adjusted R^2^ = 0.829) (Fig 2A). We verified the equation and coefficient K in the validation cohort. There was a linear correlation between the calculated TBMM and DXA measured TBMM (*P* < .001, adjusted R^2^ = 0.856, β = 1.03) (Fig 2B). By comparison, the calculated TBMM by the novel equation could explain DXA measured TBMM by 85.6%, although individual body weight could explain TBMM by 69.6%. We graphically compared the calculated TBMM and DXA measured TBMM with a Bland-Altman plot (Fig 3), and agreements between the two techniques were measured by intraclass correlation coefficients (ICC). ICC in validation cohort was 0.93 (*P* < .001) and ICC in derivation cohort was 0.91 (*P* < .001) respectively, which showed statistical agreements between two methods in both cohorts (Fig 3).

## Discussion

In the current study, eGFR adjusted serum creatinine level was proportional to the TBMM, and eGFR adjusted serum cystatin C level was proportional to total-body fat percent. We then made a novel equation to estimate TBMM and determined the coefficient of the equation in the derivation cohort. We performed verification in the validation cohort, and found that the novel equation can estimate TBMM with statistical significance. Furthermore, the errors of the equation were statistically acceptable.

Cystatin C is anticipated as a potential replacement for serum creatinine in GFR estimation. Cystatin C may be more useful to estimate GFR in older adults with decreased muscle mass, because previously existing formulas that predict GFR take into account gender, age, and weight, but not muscle mass. On the other hand, the cystatin C level is useful to assess the renal function in individuals with higher muscle mass as well; therefore, muscle mass can account for a large portion of the error in creatinine-based GFR estimation.[28]

In our equation, a higher cystatin C level was associated with lower muscle mass and it connotes a large fat percent. Many studies reported that there is a graded association between higher BMI and elevated serum cystatin C.[29–31] Moreover, cystatin C gene expression and secretion by adipose tissue were increased two- to threefold in obese individuals, and it was confirmed that increased production of cystatin C was contributed from enlarged adipose tissue in vitro study.[32] Hypertension, coronary heart disease, increased inflammatory marker, and low functional status were correlated with high cystatin C levels and these morbidities were also associated with the sarcopenia.[33] Furthermore, higher cystatin C was associated with frailty and poor physical function in the elderly and the association was separate from eGFR.[34] In the Health ABC study, there was an inverse correlation between eGFR and physical performance in the subgroup whose eGFR by creatinine was above 60mL/min/1.73m^2^.[35] Additionally, cystatin C was a strong independent risk factor for mortality in that study.[36] Therefore, we can make inferences that decreased muscle mass and increased fat mass elevate serum cystatin C level, and high cystatin C levels are associated with functional decline, morbidity, and mortality in sarcopenic patients.

Our study has several strengths. To the best of our knowledge, it is the first study to measure body muscle mass using serum creatinine and cystatin C levels. We proposed a novel equation to estimate TBMM, which can predict TBMM with statistical significance. Our novel equation could be used to measure body muscle mass with less cost compared to MRI, CT, and DXA without radiation hazard. Unlike MRI, CT and DXA, blood sampling can be done anywhere, and a lot of samples can be handled at the same time, therefore it can ease off the pressure on time and space. Moreover, TBMM can be calculated using archived samples of serum in large scale cohorts so we can estimate participant’s muscle status in the past. And it is useful to check patient’s temporal change by repetitive measurements. Furthermore, considering that dosing of some medications by a patient’s muscle mass might be helpful, and serum cystatin C level can predict serum levels of drugs such as vancomycin better than serum creatinine, our equation can be used in adjusting drug doses which mainly distribute in the muscle.[37, 38]

However, our study has several limitations. First, our estimation is limited to TBMM while sarcopenia has been diagnosed with decreased appendicular muscle mass. As serum creatinine and cystatin C are affected by TBMM and total-body fat mass, calculated TBMM with these markers includes non-appendicular muscle mass. However, it has been known that TBMM has a strong correlation with appendicular skeletal muscle mass.[23] Second, we regarded the TBMM measured with DXA as a reference standard. DXA cannot detect intramuscular fat infiltration which is known to affect quality of skeletal muscle, consequently we assumed in this study that muscle mass is bone mass subtracted from lean mass. Third, the correlation between cystatin C and body fat percent is lower than that of creatinine and TBMM. Previous studies showed that various clinical factors including diabetes, thyroid function, and hemoglobin.[18, 30] Although we could not collect whole participants’ clinical factors and they can be confounders of that relationship, statistical significance of the correlation between cystatin C and body fat percent was remained after adjustment of eGFR. Finally, our models were developed in a retrospective cross-sectional dataset from a single hospital in Korea and the measurements were done at only one point in time. Furthermore, people included in this study were ambulatory, who can visit hospital to take health examination. However, the present study is rather hypothesis generating and following studies in other ethnic groups, sick or frail patients, and longitudinal cohorts with larger scale are warranted for the generalization of findings in this study.

In conclusion, a new equation using serum creatinine and cystatin C levels can estimate TBMM in community-dwelling Koreans, without radiation hazard. This equation may be used to calculate muscle mass in various setting including retrospective cohort in which performing other measurement methods for muscle mass are impossible.

## References

[1] Inouye SK, Studenski S, Tinetti ME, Kuchel GA. Geriatric syndromes: clinical, research, and policy implications of a core geriatric concept. J Am Geriatr Soc 2007;55(5):780–91. 10.1111/j.1532-5415.2007.01156.x . 17493201 PMC2409147

[2] Cawthon PM, Marshall LM, Michael Y, Dam TT, Ensrud KE, Barrett-Connor E, et al Frailty in older men: prevalence, progression, and relationship with mortality. J Am Geriatr Soc 2007;55(8):1216–23. 10.1111/j.1532-5415.2007.01259.x . 17661960

[3] Oterdoom LH, Gansevoort RT, Schouten JP, de Jong PE, Gans RO, Bakker SJ. Urinary creatinine excretion, an indirect measure of muscle mass, is an independent predictor of cardiovascular disease and mortality in the general population. Atherosclerosis. 2009;207(2):534–40. 10.1016/j.atherosclerosis.2009.05.010 . 19535078

[4] Srikanthan P, Karlamangla AS. Muscle mass index as a predictor of longevity in older adults. Am J Med 2014;127(6):547–53. 10.1016/j.amjmed.2014.02.007 . 24561114 PMC4035379

[5] Cruz-Jentoft AJ, Baeyens JP, Bauer JM, Boirie Y, Cederholm T, Landi F, et al Sarcopenia: European consensus on definition and diagnosis: Report of the European Working Group on Sarcopenia in Older People. Age ageing. 2010;39(4):412–23. 10.1093/ageing/afq034 . 20392703 PMC2886201

[6] Chen LK, Liu LK, Woo J, Assantachai P, Auyeung TW, Bahyah KS, et al Sarcopenia in Asia: consensus report of the Asian Working Group for Sarcopenia. J Am Med Dir Assoc 2014;15(2):95–101. 10.1016/j.jamda.2013.11.025 . 24461239

[7] Heymsfield SB, Arteaga C, McManus C, Smith J, Moffitt S. Measurement of muscle mass in humans: validity of the 24-hour urinary creatinine method. Am J Clin Nutr 1983;37(3):478–94. . 6829490 10.1093/ajcn/37.3.478

[8] Proctor DN, O'Brien PC, Atkinson EJ, Nair KS. Comparison of techniques to estimate total body skeletal muscle mass in people of different age groups. Am J Physiol 1999;277(3):E489–95. . 10484361 10.1152/ajpendo.1999.277.3.E489

[9] Engstrom CM, Loeb GE, Reid JG, Forrest WJ, Avruch L. Morphometry of the human thigh muscles. A comparison between anatomical sections and computer tomographic and magnetic resonance images. J Anat 1991;176:139–56. . 1917669 PMC1260321

[10] Mitsiopoulos N, Baumgartner RN, Heymsfield SB, Lyons W, Gallagher D, Ross R. Cadaver validation of skeletal muscle measurement by magnetic resonance imaging and computerized tomography. J Appl Physiol 1998;85(1):115–22. . 9655763 10.1152/jappl.1998.85.1.115

[11] Heymsfield SB, Smith R, Aulet M, Bensen B, Lichtman S, Wang J, et al Appendicular skeletal muscle mass: measurement by dual-photon absorptiometry. Am J Clin Nutr 1990;52(2):214–8. . 2375286 10.1093/ajcn/52.2.214

[12] Janssen I, Heymsfield SB, Baumgartner RN, Ross R. Estimation of skeletal muscle mass by bioelectrical impedance analysis. J Appl Physiol 2000;89(2):465–71. . 10926627 10.1152/jappl.2000.89.2.465

[13] Chien M-Y, Huang T-Y, Wu Y-T. Prevalence of Sarcopenia Estimated Using a Bioelectrical Impedance Analysis Prediction Equation in Community-Dwelling Elderly People in Taiwan. J Am Geriatr Soc 2008;56(9):1710–5. 10.1111/j.1532-5415.2008.01854.x . 18691288

[14] Kyle UG, Bosaeus I, De Lorenzo AD, Deurenberg P, Elia M, Manuel Gomez J, et al Bioelectrical impedance analysis-part II: utilization in clinical practice. Clin Nutr 2004;23(6):1430–53. 10.1016/j.clnu.2004.09.012 . 15556267

[15] Trutschnigg B, Kilgour RD, Reinglas J, Rosenthall L, Hornby L, Morais JA, et al Precision and reliability of strength (Jamar vs. Biodex handgrip) and body composition (dual-energy X-ray absorptiometry vs. bioimpedance analysis) measurements in advanced cancer patients. Appl Physiol Nutr Metab 2008;33(6):1232–9. 10.1139/H08-122 . 19088782

[16] Levey AS, Bosch JP, Lewis JB, Greene T, Rogers N, Roth D. A more accurate method to estimate glomerular filtration rate from serum creatinine: a new prediction equation. Modification of Diet in Renal Disease Study Group. Ann Intern Med 1999;130(6):461–70. 10.7326/0003-4819-130-6-199903160-00002 . 10075613

[17] Levey AS, Stevens LA, Schmid CH, Zhang YL, Castro AF 3rd, Feldman HI, et al A new equation to estimate glomerular filtration rate. Ann Intern Med 2009;150(9):604–12. 10.7326/0003-4819-150-9-200905050-00006 . 19414839 PMC2763564

[18] Stevens LA, Schmid CH, Greene T, Li L, Beck GJ, Joffe MM, et al Factors other than glomerular filtration rate affect serum cystatin C levels. Kidney Int 2009;75(6):652–60. 10.1038/ki.2008.638 . 19119287 PMC4557800

[19] Goldberg TH, Finkelstein MS. Difficulties in estimating glomerular filtration rate in the elderly. Arch Intern Med 1987;147(8):1430–3. 10.1001/archinte.1987.00370080066014 . 3453695

[20] Coll E, Botey A, Alvarez L, Poch E, Quinto L, Saurina A, et al Serum cystatin C as a new marker for noninvasive estimation of glomerular filtration rate and as a marker for early renal impairment. Am J kidney Dis 2000;36(1):29–34. 10.1053/ajkd.2000.8237 . 10873868

[21] Schaeffner ES, Ebert N, Delanaye P, Frei U, Gaedeke J, Jakob O, et al Two novel equations to estimate kidney function in persons aged 70 years or older. Ann Intern Med 2012;157(7):471–81. 10.7326/0003-4819-157-7-201210020-00003 . 23027318

[22] Dharnidharka VR, Kwon C, Stevens G. Serum cystatin C is superior to serum creatinine as a marker of kidney function: a meta-analysis. Am J kidney Dis 2002;40(2):221–6. 10.1053/ajkd.2002.34487 . 12148093

[23] Kim J, Wang Z, Heymsfield SB, Baumgartner RN, Gallagher D. Total-body skeletal muscle mass: estimation by a new dual-energy X-ray absorptiometry method. Am J Clin Nutr 2002;76(2):378–83. . 12145010 10.1093/ajcn/76.2.378

[24] Chew-Harris JS, Florkowski CM, George PM, Elmslie JL, Endre ZH. The relative effects of fat versus muscle mass on cystatin C and estimates of renal function in healthy young men. Ann Clin Biochem 2013;50(Pt 1):39–46. 10.1258/acb.2012.011241 . 23129724

[25] Horber FF, Gruber B, Thomi F, Jensen EX, Jaeger P. Effect of sex and age on bone mass, body composition and fuel metabolism in humans. Nutrition 1997;13(6):524–34. 10.1016/S0899-9007(97)00031-2 . 9263233

[26] Bland JM, Altman DG. Statistical methods for assessing agreement between two methods of clinical measurement. Lancet 1986;1(8476):307–10. 10.1016/S0140-6736(86)90837-8 . 2868172

[27] Inker LA, Schmid CH, Tighiouart H, Eckfeldt JH, Feldman HI, Greene T, et al Estimating glomerular filtration rate from serum creatinine and cystatin C. N Engl J Med 2012;367(1):20–9. 10.1056/NEJMoa1114248 . 22762315 PMC4398023

[28] Baxmann AC, Ahmed MS, Marques NC, Menon VB, Pereira AB, Kirsztajn GM, et al Influence of muscle mass and physical activity on serum and urinary creatinine and serum cystatin C. Clin J Am Soc Nephrol 2008;3(2):348–54. 10.2215/CJN.02870707 . 18235143 PMC2390952

[29] Muntner P, Winston J, Uribarri J, Mann D, Fox CS. Overweight, obesity, and elevated serum cystatin C levels in adults in the United States. Am J Med 2008;121(4):341–8. 10.1016/j.amjmed.2008.01.003 . 18374694 PMC3049932

[30] Knight EL, Verhave JC, Spiegelman D, Hillege HL, de Zeeuw D, Curhan GC, et al Factors influencing serum cystatin C levels other than renal function and the impact on renal function measurement. Kidney Int 2004;65(4):1416–21. 10.1111/j.1523-1755.2004.00517.x . 15086483

[31] Kottgen A, Selvin E, Stevens LA, Levey AS, Van Lente F, Coresh J. Serum cystatin C in the United States: the Third National Health and Nutrition Examination Survey (NHANES III). Am J kidney Dis 2008;51(3):385–94. 10.1053/j.ajkd.2007.11.019 . 18295054

[32] Naour N, Fellahi S, Renucci JF, Poitou C, Rouault C, Basdevant A, et al Potential contribution of adipose tissue to elevated serum cystatin C in human obesity. Obesity 2009;17(12):2121–6. 10.1038/oby.2009.96 . 19360013

[33] Wasén E, Isoaho R, Mattila K, Vahlberg T, Kivelä S-L, Irjala K. Serum cystatin C in the aged: relationships with health status. Am J Kidney Dis 2003;42(1):36–43. 10.1016/s0272-6386(03)00406-2 . 12830454

[34] Hart A, Paudel ML, Taylor BC, Ishani A, Orwoll ES, Cawthon PM, et al Cystatin C and frailty in older men. J Am Geriatr Soc 2013;61(9):1530–6. 10.1111/jgs.12413 . 24001352 PMC3773269

[35] Odden MC, Chertow GM, Fried LF, Newman AB, Connelly S, Angleman S, et al Cystatin C and measures of physical function in elderly adults: the Health, Aging, and Body Composition (HABC) Study. Am J Epidemiol 2006;164(12):1180–9. 10.1093/aje/kwj333 . 17035344

[36] Shlipak MG, Wassel Fyr CL, Chertow GM, Harris TB, Kritchevsky SB, Tylavsky FA, et al Cystatin C and mortality risk in the elderly: the health, aging, and body composition study. J Am Soc Nephrol 2006;17(1):254–61. 10.1681/ASN.2005050545 . 16267155

[37] Lake KD, Peterson CD. A Simplified Dosing Method for Initiating Vancomycin Therapy. Pharmacotherapy 1985;5(6):340–4. 10.1002/j.1875-9114.1985.tb03441.x . 4080569

[38] Frazee EN, Rule AD, Herrmann SM, Kashani KB, Leung N, Virk A, et al Serum cystatin C predicts vancomycin trough levels better than serum creatinine in hospitalized patients: a cohort study. Crit Care 2014;18(3):R110 10.1186/cc13899 . 24887089 PMC4075252

